# Effects of the Selective Serotonin Reuptake Inhibitor Fluoxetine on Developing Neural Circuits in a Model of the Human Fetal Cortex

**DOI:** 10.3390/ijms221910457

**Published:** 2021-09-28

**Authors:** Kinsley Tate, Brenna Kirk, Alisia Tseng, Abigail Ulffers, Karen Litwa

**Affiliations:** 1 Department of Anatomy and Cell Biology, Brody School of Medicine, East Carolina University, Greenville, NC 27834, USA; ktate18@vt.edu (K.T.); brennaowens22@gmail.com (B.K.); tsenga19@students.ecu.edu (A.T.); ulffersa19@students.ecu.edu (A.U.); 2 Graduate Program in Biomedical Engineering, Department of Engineering, College of Engineering and Technology, East Carolina University, Greenville, NC 27834, USA

**Keywords:** fluoxetine, selective serotonin reuptake inhibitors, neurodevelopment, synapse, neurite, human cortical spheroids

## Abstract

The developing prenatal brain is particularly susceptible to environmental disturbances. During prenatal brain development, synapses form between neurons, resulting in neural circuits that support complex cognitive functions. In utero exposure to environmental factors such as pharmaceuticals that alter the process of synapse formation increases the risk of neurodevelopmental abnormalities. However, there is a lack of research into how specific environmental factors directly impact the developing neural circuitry of the human brain. For example, selective serotonin reuptake inhibitors are commonly used throughout pregnancy to treat depression, yet their impact on the developing fetal brain remains unclear. Recently, human brain models have provided unprecedented access to the critical window of prenatal brain development. In the present study, we used human neurons and cortical spheroids to determine whether the selective serotonin reuptake inhibitor fluoxetine alters neurite and synapse formation and the development of spontaneous activity within neural circuits. We demonstrate that cortical spheroids express serotonin transporter, thus recapitulating the early developmental expression of serotonin transporter associated with cortical pyramidal neurons. Cortical spheroids also appropriately express serotonin receptors, such as synaptic 5-HT2A and glial 5-HT5A. To determine whether fluoxetine can affect developing neural circuits independent of serotonergic innervation from the dorsal and medial raphe nuclei, we treated cortical neurons and spheroids with fluoxetine. Fluoxetine alters neurite formation in a dose-dependent fashion. Intriguingly, in cortical spheroids, neither acute nor chronic fluoxetine significantly altered excitatory synapse formation. However, only acute, but not chronic fluoxetine exposure altered inhibitory synaptogenesis. Finally, fluoxetine reversibly suppresses neuronal activity in a dose-dependent manner. These results demonstrate that fluoxetine can acutely alter synaptic function in developing neural circuits, but the effects were not long-lasting. This work provides a foundation for future studies to combine serotonergic innervation with cortical spheroids and assess the contributions of fluoxetine-induced alterations in serotonin levels to brain development.

## 1. Introduction

Synapses are the basis of information transfer within neural circuits, underlying the development of complex cognitive functions [1,2]. Synapses form during mid-fetal gestation between pre-synaptic axon terminals and the post-synaptic dendritic shaft or filopodia-like projections that emanate from the dendritic shaft [3,4,5]. Later in development, excitatory synapses shift to specialized dendritic spines [6]. In humans, numerous neurodevelopmental disorders manifest with altered spine morphologies and densities [7,8,9]. Both genetic factors and exposure to environmental toxicants contribute to the emergence of neurodevelopmental disorders, such as autism spectrum disorders [10]. Whether maternal use of antidepressants such as selective serotonin reuptake inhibitors (SSRIs) contributes to the development of autism spectrum disorders in offspring has been intensely debated [11]. SSRIs have been shown to disrupt normal cognitive function in rodents and may induce deficiencies in social behaviors similar to those displayed by individuals with autism [12,13]. In humans, epidemiological studies have found that in utero SSRI exposure increases the risk of developing an autism spectrum disorder, particularly when exposure occurs during the second or third trimester [14,15,16]. Notably, this period coincides with synapse formation. However more recent studies accounting for the cofounding factor of maternal psychiatric illness have found no association between SSRIs and the development of autism in offspring [17,18]. Thus, basic research is needed to address whether SSRI exposure can alter synapse formation and developing neural circuits of the fetal brain.

In the present study, we assess whether fluoxetine, an SSRI frequently used to treat pregnant woman suffering from depression [19,20], is capable of altering developing cortical neural circuits independent of serotonergic innervation. While the serotonergic system is one of the earliest neurotransmitter systems to develop in the fetal brain, serotonergic neurons emanate from the dorsal and medial raphe nuclei of the brainstem [21]. Serotonergic neurons innervate most areas of the brain, including the developing cerebral cortex [21]. Serotonin, also known as 5-hydroxytryptamine (5-HT), contributes to many key processes in the brain including emotional states, memory and learning via modulation of synapse formation and neurogenesis [22]. The 5-HT receptor class comprises G-protein coupled receptors specific to serotonin, with the exception of 5-HT_3_, which is a ligand-gated ion channel [23]. As G-protein coupled receptors, these receptors refine developing synaptic circuits through the regulation of second messenger production. The cerebral cortex expresses the greatest density of serotonin receptors. The most highly expressed receptors in the pre-frontal cortex are 5-HT_1A_ and 5-HT_2A_ [24]. Approximately 60% of all pre-frontal pyramidal neurons contain either 5-HT_1A_ and/or 5-HT_2A_ receptors, and the majority (~80%) of these neurons contain both receptors [24]. During synaptic transmission, serotonin binds to 5-HT receptors located on the post-synaptic neuron. Excess serotonin is recycled from the synaptic cleft and transported through the serotonin reuptake transporter (SERT) located on the pre-synaptic neuron [25]. SSRIs are used in the treatment of psychiatric disorders to increase serotonin levels in the synaptic cleft by blocking SERTs [26]. However, increasing evidence suggests that SSRIs such as fluoxetine can alter neurodevelopment independent of serotonin. For example, fluoxetine inhibits voltage-gated potassium channels in non-neuronal human embryonic kidney (HEK) cells expressing Kv2.1 [27]. Additionally, fluoxetine, but not other SSRIs, alters morphogenesis in a mouse embryoid body model through inhibition of Wnt signaling [28]. Wnt signaling pathways regulate patterning of the developing brain through inhibition of glycogen synthase kinase-3 beta (GSK-3β), leading to β-catenin nuclear translocation and the expression of target genes, such as cell cycle genes, cyclin D and c-myc [29]. Consistent with fluoxetine inhibition of Wnt in neural development, fluoxetine decreased neural proliferation [29]. Thus, SSRIs, can have effects on the developing brain independent of serotonin levels, making it important to assess the impact of fluoxetine exposure on neural circuit development in the absence of serotonergic innervation. Thus, in the present study, we treated human iPSC-derived cortical neurons and spheroids with fluoxetine and analyzed the effects on neurite formation as well as synapse formation and function. While fluoxetine minimally impacted neurite formation and excitatory synaptogenesis, it did reversibly inhibit developing spontaneous activity. This research provides a foundation for future studies to distinguish between serotonin-dependent and -independent effects of SSRIs on brain development.

## 2. Results

### 2.1. Dose-Dependent Effects of Fluoxetine on Neurite Formation during Neuronal Differentiation

**Figure 1:**
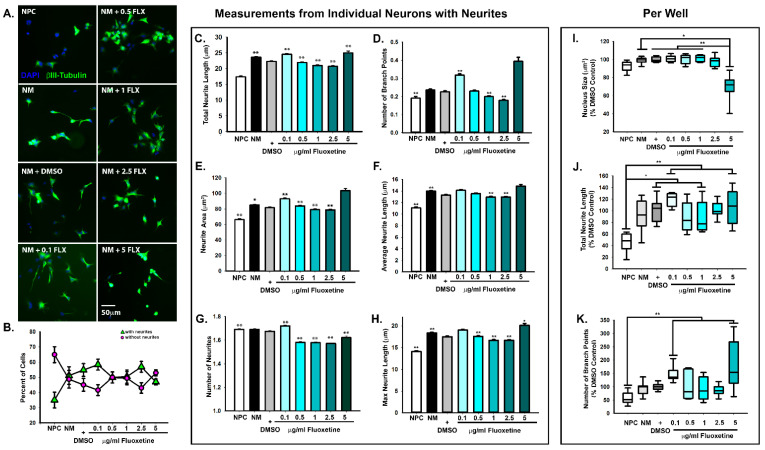
Effects of fluoxetine on neurite formation. (**A**) Human induced pluripotent stem cell derived neural progenitor cells were differentiated into neurons for 24 h in the presence of increasing concentrations of fluoxetine (FLX) before fixation and immunostaining with the neuronal marker, βIII-Tubulin and DAPI. As a control, cells were maintained in neural progenitor cell media. Cells were imaged and analyzed on the automated high content imaging system, and the experiment was performed in triplicate. Scale bar = 50 μm. (**B**) Analysis of the percentage of βIII-tubulin-positive cells with or without neurites. Note that while some NPCs express βIII-tubulin after 24 h of attachment at low-density to laminin-coated plates, the majority do not express neurites. (**C**–**H**) In the βIII-tubulin-positive cells with neurites, we analyzed total neurite length per neuron (**C**), branch points (**D**), neuron area (**E**), average neurite length (**F**), number of neurites per neuron (**G**), and max neurite length per neuron (**H**). n of βIII-tubulin-positive cells with neurites = 11,044 NPC, 15,723 NM, 18,522 DMSO, 17,224 0.1 FLX, 22,227 0.5 FLX, 23,015 1 FLX, 22,775 2.5 FLX, and 5489 5 FLX. For (**C**–**H**), data is presented as the mean + standard error. (**I**–**K**) Nucleus size and neurite parameters, including total neurite length and number of branch points, were also analyzed for the entire βIII-tubulin-positive population per well. n = 9 wells total for all conditions, except for 0.1 μg/mL, which was analyzed in 8 wells, due to low neuron count in one well. * *p* ≤ 0.05, ** *p* ≤ 0.005, one-way ANOVA. For (**C**–**H**), significant differences show for comparison with DMSO control. All comparisons reported in Supplemental Materials.

We first sought to determine whether fluoxetine alters neurite formation, which precedes the formation of synapses between neurons. When screening for pharmaceutical effects, it is important to consider multiple neurodevelopmental stages, as specific drugs may differentially impact distinct processes in the formation of neural circuits [30]. Notably, fluoxetine has been shown to upregulate neuroplasticity genes independent of serotonin [31] and to alter neurite outgrowth in non-serotonergic neural cell lines, mammalian primary neurons, and invertebrate neurons [32,33] similar to other selective serotonin reuptake inhibitors [34]. We therefore began by treating human neural progenitor cells with increasing doses of fluoxetine during the first 24 h of neural differentiation, as we have previously done to determine effects on early stages of neural differentiation and neurite extension [35,36]. The doses were selected to capture the range of clinically relevant concentrations, as brain fluoxetine concentrations are often higher than blood plasma levels, and may reach up to 10 μg/mL [37], while blood plasma levels are often around 100 ng/mL or 0.1 μg/mL [38], the lowest dose used in the present study. Notably, while these neurons have yet to assume specific identities, such as glutamatergic or GABAergic, the protocol used predominantly generates glutamatergic cortical neurons [39]. We validated the success of neuronal differentiation by comparison with neural progenitor cells, which were maintained in neural progenitor maintenance media. In comparison to cells treated with neural differentiation media, neural progenitor cells have much fewer neurites (Figure 1A,B). To analyze neurite formation in an unbiased fashion, we used an automated high content imaging and analysis system. For statistical comparison, we used the vehicle DMSO control. When we compared each neuron individually, we observed a significant increase in the number of neurites, total neurite length per neuron, neurite area, and branching at the lowest dose of 0.1 μg/mL fluoxetine, while intermediate fluoxetine doses tended to decrease neurite formation, area, length, and branching (Figure 1C–H) similar to previous reports [32,33,34]. At the highest dose of 5 μg/mL fluoxetine, we observed significant decreases in the number of neurites, but increases in their total and maximum neurite length. However, there was considerable variability in response to 5 μg/mL fluoxetine; for example, while the mean of the maximum neurite length increased, the median decreased (Figure 1H, Supplemental Materials). This variability is likely due to cytotoxic effects observed with higher fluoxetine doses [40,41,42,43]. In support of cytotoxic stress, we did observe a significant decrease in the size of the nucleus at of 5 μg/mL fluoxetine, a feature of cytotoxic stress observed during apoptotic cell death (Figure 1I) [44]. Given the large sample size obtained with automated high content imaging, statistical significance can be obtained for weak effects, with limited biological relevance [45]. Thus, we decided to compare the averages of the entire neural population within each treated well. Interestingly, when comparing neuronal populations, only the DMSO control, 0.1, 1, and 5 μg/mL fluoxetine increased total neurite length over neural progenitor cells, with the most significant increases observed for 0.1 and 5 μg/mL fluoxetine, and only 0.1 and 5 μg/mL fluoxetine increased branching when compared to neural progenitor cells (Figure 1J,K). In conclusion, most of the tested fluoxetine concentrations were not associated with adverse cytotoxic effects, nor did they robustly alter neuronal morphology, as measured by neurite length and branching (Figure 1J,K).

### 2.2. Characterization of Serotonergic Pathways in Cortical Spheroids

**Figure 2:**
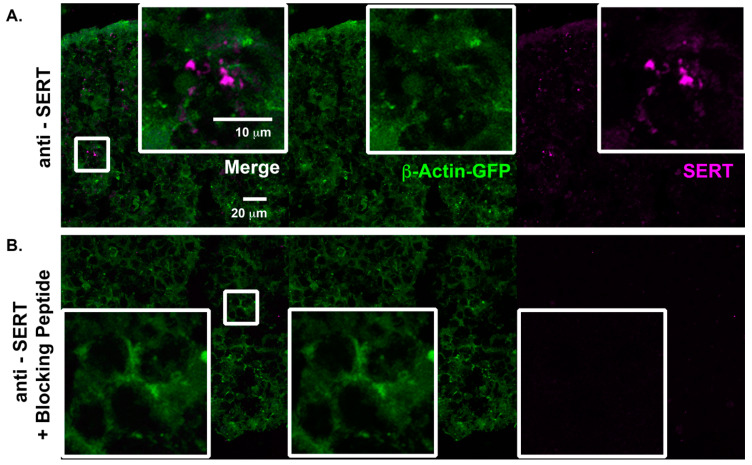
Human cortical spheroids express the serotonin transporter SERT. (**A**) 3-month-old untreated cortical spheroids were immunostained with the SERT antibody or (**B**) the SERT antibody together with the blocking peptide to evaluate antibody specificity. Endogenous β-actin-GFP (green) and immunostained SERT (magenta) were imaged with confocal microscopy under identical conditions. Scale bar = 10, 20 μm.

**Figure 3:**
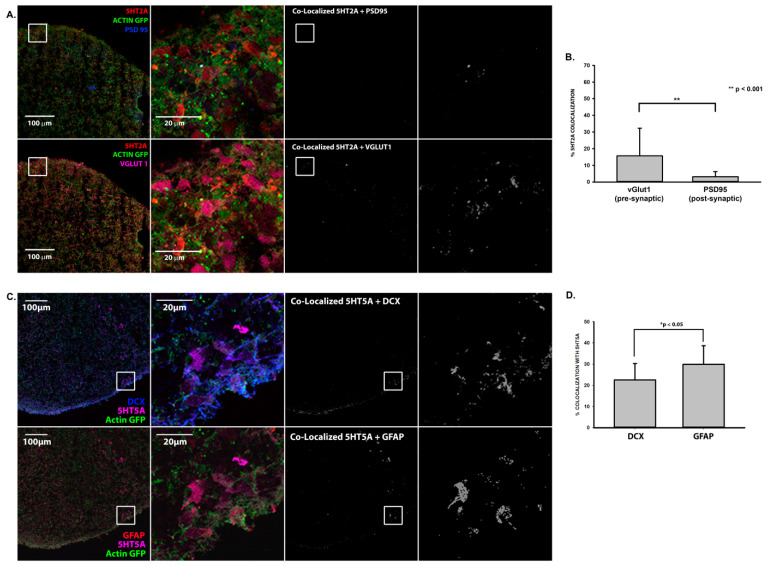
Expression of serotonin receptors in human cortical spheroids. (**A**) 3-month-old untreated cortical spheroids were immunostained for the serotonin receptor, 5-HT2A (red), and excitatory post- (PSD95, blue) and pre- (VGLUT1, magenta) synaptic markers and imaged with confocal microscopy together with endogenous β-actin-GFP. The same cryosection is shown for comparison of 5-HT2A association with PSD95 and VGLUT1. Co-localization between 5-HT2A and the respective synaptic marker is shown in grayscale. The same region of interest is enlarged for the merged and co-localized images. (**B**) Analysis of 5-HT2A colocalization with pre- and post-synaptic markers. ~20% of 5-HT2A co-localizes with pre-synaptic VGLUT1. n = 11 cryosections from 4 independent cortical spheroid cultures. (**C**) 3-month-old untreated cortical spheroids were immunostained for the serotonin receptor, 5-HT5A (magenta), and doublecortin (blue) to identify neurons and GFAP (red) to identify astrocytes and were imaged with confocal microscopy together with endogenous β-actin-GFP. The same cryosection is shown for comparison of 5-HT5A association with doublecortin (DCX) and GFAP. Co-localization between 5-HT5A and either DCX-positive neurons or GFAP-positive astrocytes is shown in grayscale. The same region of interest is enlarged for the merged and co-localized images. (**D**) Analysis of DCX-positive neurons or GFAP-positive astrocytes co-localized with 5-HT5A. Approximately 30% of GFAP co-localizes with 5-HT5A, significantly more than the ~20% of DCX positive neurons. n = 12 cryosections from 4 independent cortical spheroid cultures. Scale bar = 100, 20 μm.

We have previously demonstrated that human cortical spheroids are an ideal model to observe and manipulate synapse formation of developing neural networks [3,36,46,47]. Before we treated cortical spheroids with fluoxetine, we examined whether cortical spheroids appropriately express SERT, which is targeted by fluoxetine, and serotonin receptors, independent of serotonergic innervation from the dorsal and medial raphe nuclei. Embryonic and early post-natal brain development is associated with transient forebrain expression of SERT in cortical pyramidal neurons [48,49,50]. We have previously demonstrated that human cortical spheroids capture the fetal localization of other signaling pathways, such as endocannabinoid pathways [47]. To determine whether cortical spheroids capture the early developmental-specific expression of SERT, we immunostained for SERT in three-month-old human cortical spheroids (Figure 2A). To verify the specificity of SERT immunostaining, we also immunostained in the presence of the soluble peptide antigen, which prevented fluorescent labeling of the sample (Figure 2B), demonstrating specificity of the SERT antibody. We also examined whether common cortical serotonergic receptors exhibit an appropriate localization. The serotonergic receptor 5-HT2A is one of the most highly expressed serotonergic receptors of the cortex, where It localizes to excitatory synapses [24]. 5-HT2A co-localized with VGLUT1 and PSD95 in excitatory synapses, although it predominantly localized to pre-synaptic VGLUT1-positive compartments (Figure 3A,B). Furthermore, the serotonin receptor 5-HT5A, is preferentially expressed by glial cells (Figure 3C,D), as has been previously described in the rodent cortex [51]. Thus, despite lacking serotonergic innervation, cortical spheroids appropriately express SERT and serotonin receptors.

### 2.3. Effects of Fluoxetine on Synapse Formation

**Figure 4:**
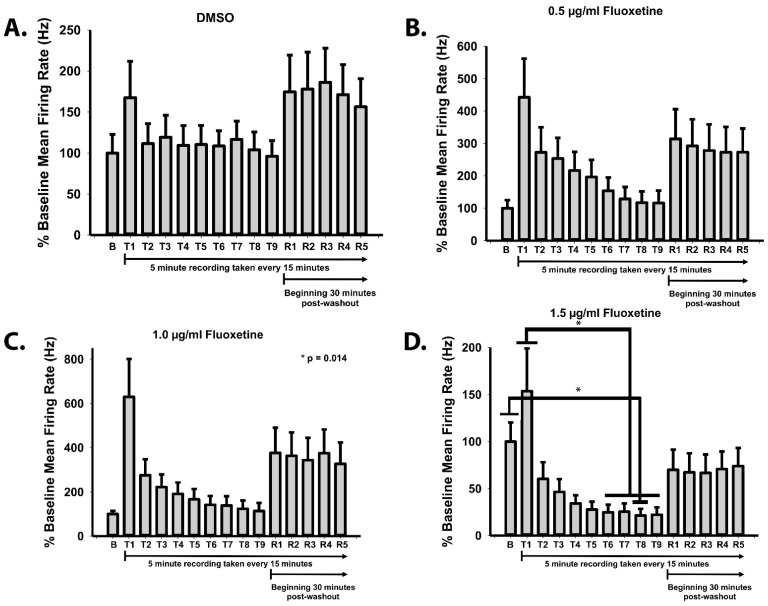
Fluoxetine reversibly suppresses spontaneous action potentials. Multielectrode array recordings of human cortical spheroids dissociated onto MEA plates. A baseline recording (labeled B) was measured to determine the normal firing rate before adding fresh media with either DMSO solvent control or increasing concentrations of fluoxetine. Neural activity was recorded every 15 min for 5 min for a total of 2 h (T1-T9). After 2 h, the drug was washed out with fresh neuronal media and after 30 min 5 recovery recordings (R1-R5) were taken every 15 min for 5 min each. (**A**) DMSO does not significantly alter firing rate. (**B**) 0.5 μg/mL FLX does not alter firing rate. (**C**) 1 μg/mL FLX does not alter firing rate. (**D**) 1.5 μg/mL FLX significantly decreases firing rate from baseline and the initial spike in activity in response to fresh media between T6-T9. n = 12 independent MEA wells from 2 independent human cortical spheroid cultures. * *p* < 0.05, One-way ANOVA.

**Figure 5:**
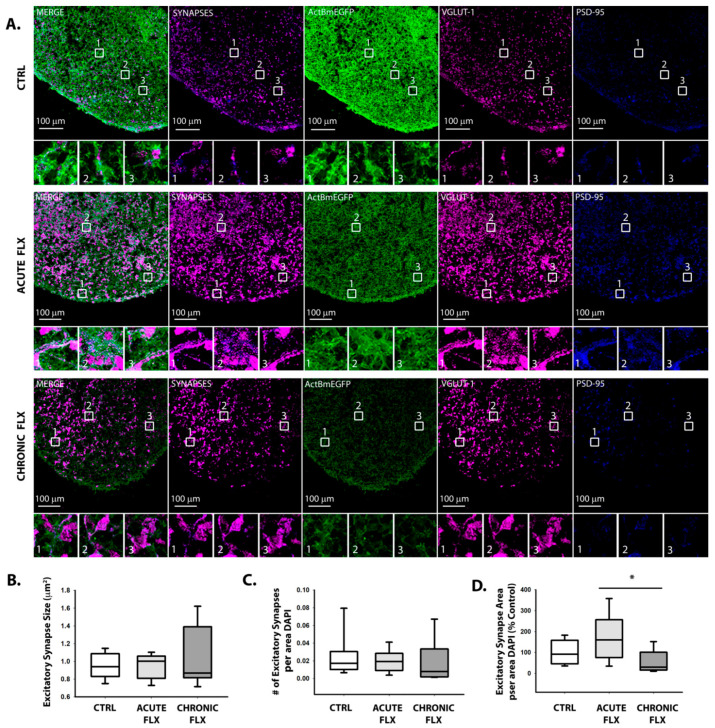
Fluoxetine does not alter excitatory synapse formation. (**A**) 3-month-old cortical spheroids were either acutely or chronically treated with 1.5 μg/mL fluoxetine. 10 μm-thick cryosections were immunostained for excitatory synapse markers, pre-synaptic VGLUT1 (magenta) and post-synaptic PSD95 (blue). Synapses were imaged together with endogenous β-actin-GFP (green) using confocal microscopy. Scale bar = 100 μm. (**B**–**D**) Co-localization between pre- and post-synaptic markers was analyzed to determine the size and density of excitatory synapses per cryosection. A threshold of 0.001 synapses per μm^2^ DAPI was used for inclusion in the study. n = 9 cryosection regions from 4 independent cortical spheroid cultures for the untreated control, 7 cryosection regions from 3 independent cortical spheroid cultures for acute FLX, and 9 cryosection regions from 3 independent cortical spheroid cultures for chronic FLX. * *p* < 0.05, One-way ANOVA.

To determine the effects of fluoxetine on synapse formation, human-derived cortical spheroids were either acutely or chronically treated with fluoxetine. For acute treatment, 1.5 μg/mL fluoxetine was added overnight to three-month-old cortical spheroids. We have previously shown that acute pharmacological treatment is sufficient to alter synapse formation [36,46,47]. In contrast, for chronic treatment, fluoxetine was added for a month and half, when cortical spheroids are being cultured in neuronal maintenance media so as to not interfere with preceding events of neural differentiation and migration. We chose the concentration of 1.5 μg/mL fluoxetine given that this dose alters synapse function (Figure 4), but does not produce cytotoxic effects, such as those observed at 5 μg/mL fluoxetine (Figure 1). After three months of culture, the cortical spheroids were cryosectioned and immunostained for excitatory synaptic markers, VGLUT1 and PSD95. To identify excitatory synapses, we analyzed co-localization of the pre-synaptic marker VGLUT1 and post-synaptic PSD-95 (Figure 5A). There were no significant changes in excitatory synapse formation in comparison to control spheroids, although there was a significant difference in the area occupied by excitatory synapses in acute vs. chronically treated spheroids, with acute spheroids exhibiting more excitatory synapse area (Figure 5D). Thus, while we observed a trend towards an increased area occupied by excitatory synapses in cortical spheroids acutely treated with fluoxetine (Figure 5D), there were no significant changes in the size of individual excitatory synapses (Figure 5B) or density of excitatory synapses (Figure 5C).

**Figure 6:**
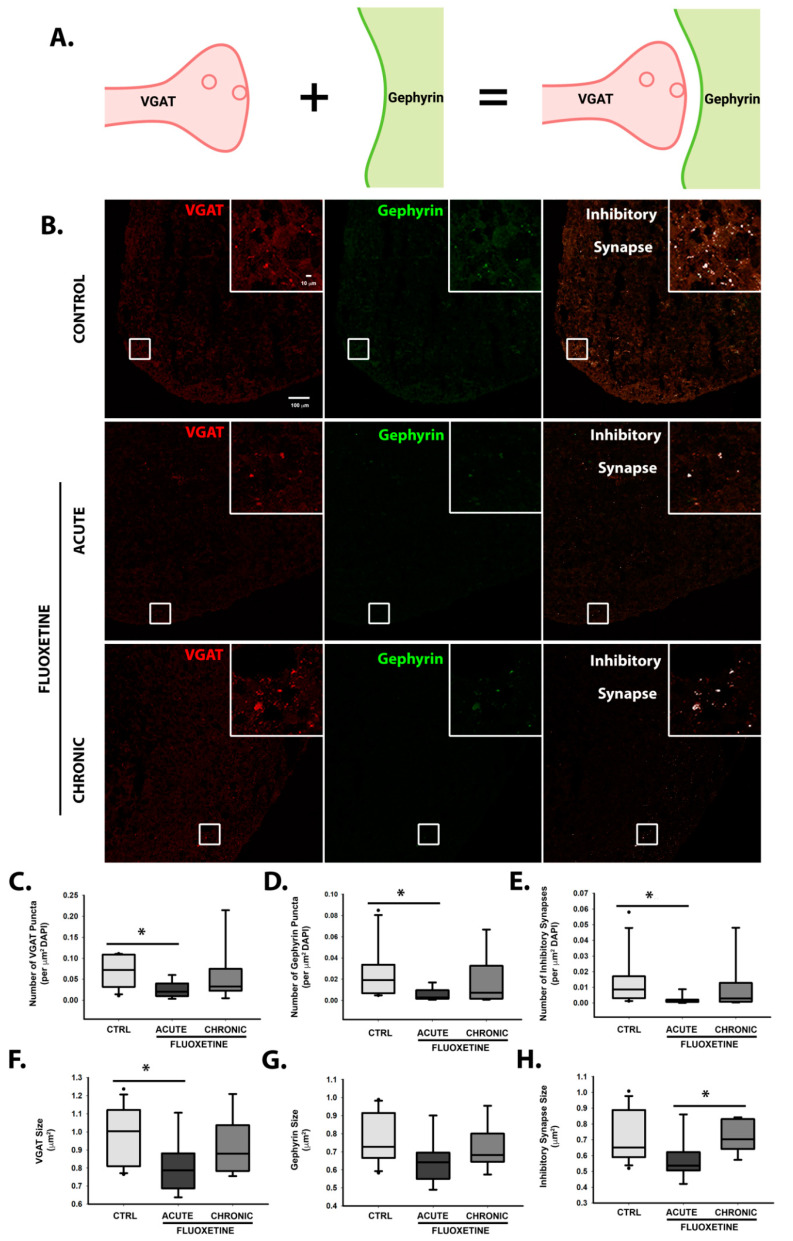
Acute, but not chronic, fluoxetine alters inhibitory synapse formation. (**A**) Schematic of inhibitory pre- (VGAT-positive) and post- (gephyrin-positive) synaptic compartments. Image created using lab-licensed Biorender account. (**B**) 3-month-old cortical spheroids were either acutely or chronically treated with 1.5 μg/mL fluoxetine. 10 μm-thick cryosections were immunostained for inhibitory synapse markers, pre-synaptic VGAT (red) and post-synaptic gephyrin (green) and were imaged using confocal microscopy. Scale bar = 10, 100 μm. (**C**–**H**) Individual pre- (VGAT+) and post- (gephyrin+) synaptic markers, as well as co-localization between pre- and post-synaptic markers were analyzed to determine the size and density of inhibitory synapses per cryosection. A threshold of 0.001 synapses per μm^2^ DAPI was used for inclusion in the study. n = 12 cryosections from 4 independent cortical spheroid cultures for the untreated control, 9 cryosections from 3 independent cortical spheroid cultures for acute FLX, and 9 cryosections from 3 independent cortical spheroid cultures for chronic FLX. * *p* < 0.05, One-way ANOVA.

While cortical spheroids recapitulate dorsal forebrain development and predominantly express glutamatergic neurons [52,53], we observe a smaller portion of inhibitory synapses [46,47]. We therefore assessed whether fluoxetine alters inhibitory synaptogenesis in developing cortical spheroids by examining pre-synaptic VGAT and post-synaptic gephyrin (Figure 6A,B). Acute but not chronic fluoxetine significantly decreased the density and size of inhibitory synapses (Figure 6E,H). These effects were also observed separately for size and density of VGAT-positive pre-synaptic puncta (Figure 6C,F) and the density of gephyrin-positive post-synaptic puncta (Figure 6D,G). Intriguingly, these effects were lost with chronic treatment, suggesting compensatory mechanisms for restored neural function with continued fluoxetine exposure.

### 2.4. Fluoxetine Reversibly Suppresses Spontaneous Action Potential Formation

To determine the effects of fluoxetine on electrical neuronal activity, microelectrode array (MEA) technology was used to record the effects of the drug. For this portion of the project, the Maestro Edge (Axion BioSystems) was used to record the changes in electrical field potentials corresponding to spontaneous action potentials. Prior to experimentation, a baseline recording was taken to establish the control rate of spontaneous action potential firing. Following the baseline recording, the solvent DMSO and three concentrations of fluoxetine (0.5 μg/mL, 1.0 μg/mL, and 1.5 μg/mL) were individually added to each row on the 24-well MEA plate and the effects on spontaneous action potential firing rate were recorded for 5 min every 15 min for 2 h. Following the two-hour recording period, the drug was washed out. We initiated a new recording schedule 30 min post-washout. We analyzed mean firing rate, which corresponds to the number of spikes that occur over the duration of the 5-min recording. Notably, the addition of fresh media with treatment results in an initial spike in activity. For the DMSO control, the mean firing rate was not found to be significantly different during any of the treatment or recovery recordings when compared to baseline (Figure 4A). Apart from the initial spike in activity, neither treatment with 0.5 nor 1 μg/mL fluoxetine significantly impacted the mean firing rate (Figure 4B,C). However, 1.5 μg/mL fluoxetine significantly decreased the mean firing rate (Figure 4D), although this concentration did not impact excitatory synapse formation (Figure 4). The decrease in firing rate was reversible, and the normal firing rate was restored with the drug washout (Figure 4D).

## 3. Discussion

Autism spectrum disorders (ASD) have become increasingly more prevalent in recent years. In 2000, 1 in 150 children in the United States were diagnosed with ASD [34]. This number has since increased and was reported in 2012 as one in 68 children being diagnosed with the disorder [34]. The increased prevalence in ASD is thought to result from a combination of environmental and genetic factors. Complex interactions between these factors are difficult to study. Due to the complexity of these interactions and the genetic heterogeneity associated with ASD, patient-specific models are necessary to account for environmental factors contributing to Autism pathology. In this project, human cortical spheroids were used to model fetal brain development and examine the effects of the SSRI fluoxetine on synapse formation.

Fluoxetine is a SSRI that increases the amount of serotonin available at the synaptic cleft. This increase in serotonin leads to increased activation of serotonin receptors. However, fluoxetine also regulates neural physiology independent of serotonin, by inhibiting Wnt [28] and the potassium channel, Kv2.1 [27]. Similar to what is observed in autism, fluoxetine has previously been shown to increase dendritic spine formation [14,15,16,17].To test the hypothesis that fluoxetine alters fetal synapse formation, we developed human cortical spheroids that recapitulate the second trimester fetal brain, when synapses form [3,52]. However, neither acute nor chronic fluoxetine treatment significantly altered excitatory synapse formation, and only acute but not chronic fluoxetine exposure altered inhibitory synapse formation, suggesting that compensatory mechanisms restore normal synapse formation in the presence of chronic fluoxetine administration. Intriguingly, chronic fluoxetine has been shown to promote neuroplasticity through increased BDNF [54,55], which could contribute to the observed recovery. Similarly, while fluoxetine suppressed spontaneous action potentials, this effect was reversible with the removable of fluoxetine. The minimal impact of fluoxetine to developing neural circuits is consistent with epidemiological data that fluoxetine does not significantly increase the risk of developing an autism spectrum disorder [17,18], and is consistent with data demonstrating that fluoxetine promotes juvenile-like neuroplasticity [56]. However, it should be noted that this study only considered the effects of sub-toxic fluoxetine concentrations on synapse formation and function. In both neurons and cancer cell models, increasing fluoxetine doses can induce cell stress, growth arrest, and ultimately cell death.

Since human cortical spheroids lack serotonergic innervation form the dorsal and medial raphe nuclei of brainstem [24], our research establishes a baseline for non-serotonergic effects on developing neural circuits of the cortex. Importantly, cortical spheroids appropriately express SERT and serotonin receptors (Figure 2 and Figure 3) independent of serotonergic innervation and are thus ready to respond to serotonin. The development of serotonin neurons from human induced pluripotent stem cells [57,58] makes it possible to combine them with cortical spheroids to assess how fluoxetine-induced alterations in serotonin levels impact neural circuits. Our results establish human cortical spheroids as a powerful model to address how environmental factors alter developing neural circuitry, with the ability to study complex drug effects on specific brain regions and in combination with different brain regions.

## 4. Materials and Methods

### 4.1. Cell Lines

Control WTC-11-ActBmeGFP induced pluripotent stem cells were obtained under MTA from the Coriell institute (Camden, NJ, USA). The parental WTC-11 iPSC line was developed by Bruce Conklin of the Gladstone Institute and was further gene edited by the Allen Institute for Cell Science using CRISPR/Cas9 to tag endogenous β-actin with monomeric Green Fluorescent Protein [59]. hiPSCs were maintained in Essential 8 Medium + E8 supplement (Gibco/Thermo Fisher Scientific, Waltham, MA, USA) on hESC Matrigel (Corning, NY, USA) coated plates. Upon splitting, 10 μM of the ROCK inhibitor Y27632 (Selleck Chemicals, Houston, TX, USA) was added to the cell medium. Once a month, the cultures were evaluated to ensure they were free of mycoplasma.

### 4.2. Neural Progenitor Cell Culture

Neural progenitor cells (NPCs) were generated from hiPSCs through an embryoid body protocol [39]. In summary, Dispase-II Solution was used to dissociate hiPSCs and plate them into ultra-low attachment 6-well plastic plates (Corning) to form free-floating embryoid bodies. Floating embryoid bodies were then cultured in Essential 6 Medium (Gibco/Thermo Fisher Scientific) for one day. The next day, media was switched to Dulbecco’s Modified Eagle Medium/Nutrient Mixture F-12 (DMEM/F-12) + GlutaMAX (Gibco/Thermo Fisher Scientific) supplemented with N-2 (Gibco/Thermo Fisher Scientific,), B-27 without vitamin A (Gibco/Thermo Fisher Scientific, Waltham, MA, USA), 10 μM of SB431542, and 100 nM of LDN193189 (Miltenyi Biotec, Bergisch Gladbach, Germany). Cells were cultured at 5% CO_2_ and 37 °C. After 7 days in culture, embryoid bodies were plated onto Poly-L-Ornithine (Sigma Aldrich, St. Louis, MO, USA) and laminin (Corning) -coated 6-well plastic tissue culture plates. Embryoid bodies were then cultured in the same media for an additional 7 days until neural rosettes formed. Lastly, 1 mL/well of STEMdif Neural Rosette Selection Reagent (StemCell Technologies, Vancouver, Canada) was used to dissociate neural rosettes, forming NPCs. The resulting NPCs were cultured on Poly-L-Ornithine (Sigma Aldrich) and laminin (Corning) -coated 6-well plastic tissue culture plates for the first four passages in NPC media, consisting of DMEM/F-12 + GlutaMAX (Gibco), N-2 (Gibco), B-27 without vitamin A (Gibco), 1 μg/mL of laminin (Corning) and 20 ng/mL of basic fibroblast growth factor (bFGF) (Peprotech, East Windsor, NJ, USA or Shenandoah Biotechnologies, Warminster, PA, USA). After four passages, NPCs were grown on Corning matrigel-coated 6-well plastic tissue culture plates. NPC media was replaced every other day and cells were grown to a confluency of no more than 80% before subsequent cell splitting. During cell passaging, 500 μL/well of Accutase (Gibco) was used to lift cells from plates.

### 4.3. Differentiation of hiPSC-Derived NPCs into Neurons

As we have previously described [35,36], NPCs were differentiated into neurons with neural differentiation media, consisting of DMEM/F-12 + GlutaMAX (Gibco), N-2 (Gibco), B-2 with vitamin A (Gibco), 20 ng/mL of BDNF (Shenandoah Biotechnology), 20 ng/mL of glial cell-derived neurotrophic factor (GDNF) (Shenandoah Biotechnology), 400 μM of cyclic adenosine monophosphate (cAMP) (Sigma Aldrich), and 200 nM of ascorbic acid (Fisher Scientific, Hampton, NH, USA).

### 4.4. 3-D Cortical Spheroid Culture

Cortical spheroids were produced following Pasca et al. [52]. Briefly, enzymatically lifted hiPSCs were transferred to ultra-low attachment plates and cultured in DMEM supplemented with Knockout Serum Replacement (Gibco) supplemented with 5 μM Dorsomorphin (BioVision, Milpitas, CA, USA), 10 μM SB431542 (Miltenyi Biotec) for 6 days. Then, 10 μM Y27632 (Selleck Chemicals) was added during the first 48 h. The resulting spheroids were then maintained in neurobasal media until day 90: Neurobasal A medium, 2% B-27 supplement without vitamin A, GlutaMAX (Gibco) and penicillin/ streptomycin (Gibco). Spheroids were supplemented with 20 ng/mL of FGF and EGF (PeproTech) from day 6 to 25, and 20 ng/mL of BDNF and NT3 (Shenandoah Biotechnology) from day 26 to 42. Spheroids were harvested beginning at day 90 for analysis. Once a month, the cultures were evaluated to ensure they were free of mycoplasma.

### 4.5. Drug Treatment of Cortical Spheroids

To evaluate the effects of fluoxetine on synapse formation and morphology, cortical spheroids from multiple sets were treated with the antidepressant fluoxetine (fluoxetine hydrochloride, TCI Chemicals, Tokyo, Japan, Product Number: F0750) either acutely or chronically. The chronically treated cortical spheroids were treated with fluoxetine (final concentration 1.5 μg/mL) every four days after the initial 44 days in culture. The Fluoxetine was added to the neuronal media used to regularly feed the cortical spheroids every 3–4 days. The acutely treated cortical spheroids were not treated with Fluoxetine (final concentration 1.5 μg/mL) until 24 h prior to fixation (day 89).

### 4.6. Immunohistochemistry

Post-drug treatment, cortical spheroids were fixed in 4% paraformaldehyde for 24 h and placed in 30% sucrose for 24 h. Spheroids were then embedded in OCT mounting media overnight (Sakura Finetek, Torrance, CA, USA), flash frozen, and cryosectioned into 10 μm thick sections. Cryosections were permeabilized with 0.2% TritonX-100 in 1× PBS before immunostaining. Sections were first blocked in 5% normal goat serum. Primary antibodies were diluted in 2% normal goat serum in PBS, added to fixed cultures and kept at 4 °C overnight. After three PBS washes, secondary antibodies diluted in 2% normal goat serum in PBS were added to fixed cultures and kept at room temperature for 1 h. Cryosections were mounted using Fluoro-gel II with DAPI mounting medium (Electron Microscopy Sciences, Hatfield, PA, USA) for confocal imaging. The primary antibodies used in this experiment included the pre-synaptic excitatory marker vesicular glutamate transporter 1 (VGLUT1) (Synaptic Systems Goettingen, Germany 135 304, 1:1000), the post-synaptic excitatory marker post synaptic density protein 95 (PSD-95) (Santa Cruz Biotechnology Inc, Dallsa, TX, sc-32291, 1:50), the pre-synaptic inhibitory marker vesicular GABA transporter (VGAT) (Synaptic Systems 131 004, 1:1000), and the post-synaptic inhibitory marker gephryin (Synaptic Systems 147 011C3, 1:500). Antibodies toward serotonergic proteins were also used: SERT (Alomone Lab, Jerusalem, Israel, AMT-004), SERT blocking peptide (Alomone Lab BLP-MT004), 5-HT2A (Sigma Aldrich SAB4501474) and 5-HT5A (Sigma Aldrich SAB4501483. The primary antibodies were diluted in 2% Normal Goat Serum (Vector Laboratories, Burlingame, CA, USA). The samples were incubated overnight with the primary antibody solution in a 4 °C refrigerator, followed by 3–5 min PBS washes, and incubation with secondary antibody at 1:500 in 2% Normal Goat Serum (Vector Laboratories) in PBS at room temperature for one hour. The samples were covered to prevent light from exciting the fluorophores. Post-incubation 3–5 min PBS washes were performed. The slides were rinsed with de-ionized water. A glass coverslip was affixed to the slide with Fluorogel II (Electron Microscopy Sciences) with DAPI.

### 4.7. High Content Imaging and Analysis

Fixed hiPSC-derived NPCs neurons were imaged using DAPI and βIII-Tubulin (Tuj1 clone). NPCs and neurons were imaged using the Thermo Scientific CellInsight CX5 High Content Screening Platform with a 10× objective and simultaneously analyzed using the Neuronal Profiling Assay V4.2.

### 4.8. Confocal Imaging

The immunostained samples were imaged on a Zeiss LSM 700 (Carl Zeiss, Germany) confocal microscope with a 40× Plan-Apochromat/1.4 Oil DIC M27 objective to acquire 3, 4 × 4 tile images from each slide. Each image consisted of five 0.156 μm thick slices in the z-direction. The images were subsequently analyzed using ImageJ to examine the effect of the various drug treatments on synapse formation.

### 4.9. Dissociation of Human Cortical Spheroids for MEA Experiments

After 90 days in culture, cortical spheroids were dissociated onto multi-electrode array (MEA) plates. To dissociate the cortical spheroid, they were first incubated at 37 °C for 45 min in 3 mL of a papain solution containing Earle’s balanced salts (EBSS, Sigma, E7510), d-(+)-glucose (22.5 mM), NaHCO3 (26 mM), DNase (125 U/mL, Worthington Biochemical Corporation, Lakewood, NJ, USA, LS002007), papain (30 U/mL, Worthington LS03126), and L-cysteine (1 mM, Sigma, C7880). Post-incubation the cortical spheroid was washed three times with an inhibitor buffer containing; BSA (1.0 mg/mL, Sigma A-8806) and ovomucoid (also known as trypsin inhibitor, 1.0 mg/mL, Roche Diagnostics Corporation, Indianapolis, IN, USA, Cat# 109878). The cortical spheroids were broken apart via trituration. Once the cells were dissociated, they were layered on top of high concentration inhibitor solution (5 mg/mL BSA and 5 mg/mL ovomucoid) and centrifuged for five minutes. The resulting cell pellet from centrifuging was re-suspended in Dulbecco’s phosphate-buffered saline (DPBS, Invitrogen/Thermo Fisher Scientific, Cat# 14287) with 0.02% BSA and 12.5 U/mL DNase. After the cells were adequately re-suspended, they were plated onto MEA 24-well plates. Cells were plated at a concentration of 200,000 cells per well. The plates were incubated for one hour at 37 °C. Post-incubation, 300 μL of fresh neuronal media was added to each well. The media was added carefully onto the side of each well to prevent the cells from lifting off of the electrodes. An additional 300 μL of fresh neuronal media was then added to each well, bringing the total well volume to 600 μL. The neurons were fed every four days by removing 350 μL of old media and adding 350 μL of fresh neuronal media. The cells were cultured for two weeks prior to experimentation to allow the neurons to become established on the recording electrodes.

### 4.10. MEA Plate Preparation

Prior to plating neurons from dissociated cortical spheroids, MEA plates were first treated with polyethylenimine (PEI). In a 24-well MEA plate, the bottom of each well features 16 recording electrodes. To coat the recording electrodes, a pipet tip was used to carefully apply a 10 μL droplet of PEI directly onto the electrodes. After adding a droplet of the PEI solution to each well, MEA plates were incubated for 1 h at 37 °C. Post-incubation, three washes were performed with deionized water. The plates were allowed to sit overnight in a sterile tissue culture hood to dry.

### 4.11. Maestro Edge Recordings of Neural Activity

The Maestro Edge (Axion BioSystems, Atlanta, GA, USA) incorporates multi-electrode array (MEA) technology to record electrical signals from excitable cells. This device is able to amplify electrical signaling between neurons, allowing for detailed analysis. The Maestro Edge incorporates a plate reading system that uses MEA 24-well plates. Each plate features a barcode that allows for specific recognition in the AxIS software associated with the device. Once a plate is placed inside of the Maestro Edge, the barcode is scanned, and the experimental information associated with the plate is expressed in the AxIS Navigator software. AxIS Navigator is the program used to control specific features of the Maestro Edge including incubation parameters, recording features, stimulation capabilities and signal processing. The Maestro Edge incorporates an incubation system that allows for the induction of certain environmental conditions and for prolonged electrical signal recording. For the purposes of recording electrical activity from neurons, the gain is set to 1000× and bandwidth is set to 200–4000 Hz.

### 4.12. Fluoxetine MEA Experiment

To determine the effects of fluoxetine on electrical signaling, various concentrations of fluoxetine were applied to dissociated cortical spheroids and the resulting electrical signals were recorded using the MEA technology. Prior to placing one of the plates inside of the Maestro Edge, the temperature and carbon dioxide levels necessary for incubation were calibrated to be 37 °C and 5% carbon dioxide. Once the appropriate gas and temperature levels were reached, the 24-well MEA plate was placed inside of the machine. A baseline recording was taken for 10 min. After the baseline was finished, the various treatments were prepared in a biological hood. The DMSO and Fluoxetine were diluted in neuronal media without vitamin A. Once the treatments were prepared, the MEA plate was removed from the Maestro Edge and placed in the biological hood. Then, 100 μL of media was removed from each well, bringing the total well volume to 600 μL. Subsequently, 200 μL of the various treatment medias were added to the corresponding wells. The MEA plate was placed back into the Maestro Edge. The effects of the treatments on electrical activity were recorded for two hours. A recording schedule was set up to record for five minutes, every 15 min. After the two hours, all 800 μL of media was removed from each well and 600 μL of fresh media was added. The recovery period was recorded for 1 h and 30 min. Similar to the treatment period, a recording schedule was set so a 5-min recording would be taken every 15 min.

### 4.13. Statistical Analyses

Statistical analyses were performed using SigmaPlot 13.0 (or higher) software (Systat Software, Chicago, IL, USA). One-way ANOVA tests were performed on pairwise comparisons as indicated in the figure legends to determine significance. Detailed statistical measurements are included in Supplemental Materials.

## Supplementary Materials

The following are available online at https://www.mdpi.com/article/10.3390/ijms221910457/s1.
